# 2D immunoblots show differential response of mouse IgG and IgM antibodies to antigens of mammary carcinoma 4 T1 cells

**DOI:** 10.1186/1475-2867-14-9

**Published:** 2014-01-28

**Authors:** Mariana Díaz-Zaragoza, Ricardo Hernández, Pedro Ostoa-Saloma

**Affiliations:** 1 Instituto de Investigaciones Biomédicas, Universidad Nacional Autónoma de México, Ciudad Universitaria México D.F C.P, 04510 Mexico City, Mexico; 2 Posgrado en Ciencias Biológicas, Universidad Nacional Autónoma de México, Ciudad Universitaria México D.F C.P, 04510 Mexico City, Mexico

**Keywords:** 2D Immunoblot, IgM, 4 T1 cells, Mammary carcinoma

## Abstract

**Background:**

Immunosuppression in breast cancer has been reported in women and in the highly metastatic mouse mammary tumor model 4 T1. The immunosuppressive environment complicates the use of the humoral response against the tumor as an immunodiagnostic tool. IgM has not been used in immunodiagnostic in part because its antitumor responses, both innate and adaptive, have not been studied in function of time in breast cancer.

We show a new approach to analyzing the mouse humoral immune response, and compare the evolution with time of IgG and IgM responses against the antigens of 4 T1 cells.

**Methods:**

The study is based on 2-dimensional immunoblotting detection of antigens from 4 T1 cells by the IgG and IgM antibodies in the serum of female mice injected with 4 T1 cells.

**Results:**

There was a high variability in the intra-and inter-mouse response. Variability in the IgM response was manifested as a pattern of spots that could become a multibinomial variable of 0 and 1, which could represent a signature of the immune response. Different numbers of spots was found in the IgG and IgM responses from week 1 to 5. On average, the IgM had more but the IgG response decrease with the time. The natural IgM at t = 0 responds stronger than w1; the adaptive response of both IgM and IgG were elicited where, with the former being stronger better than the latter. Antigens that are recognized by some female mice in the first week are also recognized by other female mice at time 0. Contamination of the natural IgM makes difficult use the adaptive IgM as a tool for immunodiagnostic.

**Conclusions:**

IgM and IgG response varied with the time and individuals. Spot variation in 2D pattern for the natural IgM could be expressed as a binomial signature, which opens up the way to correlate a particular pattern with resistance or susceptibility. This uncovers a battery of IgMs for each individual to confront cancer or infections. The possibility to differentiate between adaptive IgM antibodies from the natural IgM will allow investigation of the adaptive IgM for early immunodiagnosis.

## Background

During a lifetime, human beings are permanently exposed to the development of transformed cells, which arise spontaneously or by contact with trigger factors. Immune surveillance of cancer leads to the generation of anti-tumor antibodies from the early stages of the disease [1]. Once a tumor is established, however, the network of immune system associated with it, may paradoxically promote tumor growth and metastasis rather its destruction [2].The use of mouse models allows investigation of how the immunosuppression status may contribute to tumor establishment. Immunosuppression in breast cancer occurs in women [3-5], and in the highly metastatic mouse mammary tumor model 4 T1 [6,7]. An immunosuppressive environment complicates the use of the humoral response against the tumor as an immunodiagnostic tool. Autoantibodies have many attractive features as biomarkers, in particular natural IgM antibodies (Ab) are involved in the primary defense mechanism that activates the cascades of complement and apoptosis. Therefore it is one of the most important mechanisms in the detection and elimination of transformed cells as part of the innate humoral immune system [8-12]; IgMs are low-affinity antibody products of the innate immunity that detect the major changes on malignant cells as post-transcriptional modifications of carbohydrate patterns [9,13-18]. Natural IgM antibodies can induce tumor-specific apoptosis [19]. The IgM antitumor responses, both innate and adaptive, have not been studied as a function of time in breast cancer, and therefore we show a new approach for analyzing the mouse humoral immune response, and compare the evolution of IgG and IgM responses against the antigens of 4 T1 cells. The study is based on the detection of antibodies in the serum of female mice injected in the mammary gland nipple with 4 T1 cells, with analysis by two-dimensional immunoblotting. Differential behavior was found between the responses of IgG and IgM. Not only the response to IgG, but the response of IgM decreased with the time, although with different kinetics. Each mouse has an individual pattern of recognition, in our experiments to a tumor antigenic background and a variable number of spots for IgMs. In other words, each individual has a particular expression of IgMs (in number and capacity of recognition) that is genetically determined. This variability was measured by generating a signature expressed as a binomial variable. The ability to differentiate between the adaptive IgM and natural IgM antibodies allows the detection of the early antigens breast cancer in an anticipated (predictive) manner by means of immunoassay, achieving this recognizing regardless of the immunosuppressive environment.

## Results and discussion

**Figure 1:**
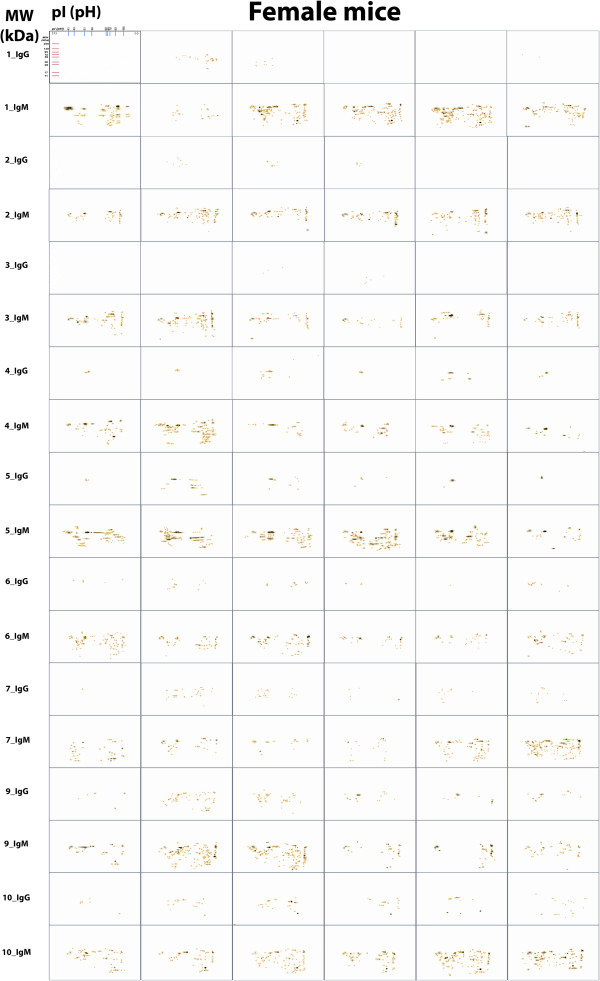
**Images of 2D-immunoblot of the immune response mediated by IgG and IgM in female mice analyzed with PdQuest software.** Images of T = 0 and w1-5 are shown. Scales of Molecular Weight (Kda) and isoelectric point (pH) are only shown in the images of 2D-immunoblot 1_IgG-T = 0. The red crosses indicate the center of each spot and the yellow circle, their area.

The images in Figure 1 of the 2D-immunoblots shows a remarkable variation among the humoral immune response mediated by IgG or IgM in each serum of individual female mice at t = 0 and during the 5 weeks of development of breast cancer. The number of antigens recognized by IgM in the first week was very similar that recognized in time 0 (518 and 563, respectively); however, the recognition pattern was different. In the analysis of IgM and IgG response with time, the Σ of IgM spots at t = 0 varied from 35 to 98 antigens, with an average of 62.6 and a standard deviation (sd) of 23.5 (Table 1). During the 5 weeks, the IgM response varied on average from 57.6 spots in the first week to 55.8 by the fifth week, with the lowest response (41 spots) in the third week. After challenge with 4 T1 tumor cells, the response IgG was, on average, the highest response at the first week (20.3 spots), but this was decreased with time. It can also be seen that 4 T1 antigen recognition by IgM is presented from week 0 (pre-immune serum), whereas there was no response of IgG (e.g. mice 1 and 2) or almost absent (e.g. mice 4 and 5). At 1 week after implantation of 4 T1 tumor cells, the IgG and IgM responses were still present; however, IgG recognition decays through to 5 weeks of tumor growth, so that the response of IgM is maintained at a higher level than IgG during tumor development. Decrease in the IgG response is possibly due to immunosuppression caused by the development of breast cancer at the time that the 4 T1 tumor cells were implanted [5]. Since the IgM response, also declined slightly in the number of spots with time, immunosuppression would also be affecting it. Decrease in the IgM response could be due to a T-independent immunosuppression, which is based on the existence of T-independent antigens as glycoproteins and glycolipids, which are recognized by natural IgM because they are capable of activating B cells without T cell collaboration [20-22]. Since the volume of serum was low, IgM could not be measured by ELISA because of even though fewer spots, the IgM concentration could be maintained, and then there would not be any immunosuppression.

**Table 1:** Number, sum, average, standard deviation and total of spots found in 2D-immunoblot images of female mice from response mediated by IgG and IgM through the development of breast cancer with 4 T1 tumor cells

**Spots IgM**								
**N° female mice**	**w0**	**w1**	**w2**	**w3**	**w4**	**w5**	**∑ spots IgM T = w1–w 5**	**TOTAL ∑ spots IgM w0 – w5**
**1**	86	20	129	83	146	75	453	539
**2**	39	77	49	49	60	89	324	363
**3**	65	66	41	18	24	31	180	245
**4**	45	94	22	24	36	15	191	236
**5**	98	69	63	91	61	13	297	395
**6**	89	21	23	23	14	23	104	193
**7**	45	28	19	14	70	144	275	320
**9**	35	120	109	17	15	43	304	339
**10**	61	23	47	46	78	69	263	324
**∑ spots by serum**	**563**	**518**	**502**	**365**	**504**	**502**	**2391**	**2954**
**Average**	**62.6**	**57.6**	**55.8**	**40.6**	**56.0**	**55.8**	**265.7**	**328.2**
**SD**	**23.6**	**36.4**	**39.0**	**29.2**	**41.4**	**43.0**	**100.1**	**102.5**
**Spots IgG**								
**N° female mice**	**w0**	**w1**	**w2**	**w3**	**w4**	**w5**	**∑ spots IgG T = w1–w 5**	**TOTAL ∑ spots IgG w0-w5**
**1**	0	27	9	0	0	2	38	38
**2**	0	8	8	5	0	0	21	21
**3**	0	0	3	5	0	0	8	8
**4**	4	3	8	2	11	5	29	33
**5**	4	34	13	2	1	1	51	55
**6**	7	10	14	5	1	10	40	47
**7**	2	30	21	5	11	12	79	81
**9**	6	51	46	15	11	15	138	144
**10**	7	20	25	17	14	21	97	104
**∑ spots by serum**	**30**	**183**	**147**	**56**	**49**	**66**	**501**	**531**
**Average**	**3.33**	**20.33**	**16.33**	**6.22**	**5.44**	**7.33**	**55.67**	**59.00**
**SD**	**2.96**	**16.73**	**13.06**	**5.85**	**6.06**	**7.55**	**41.53**	**43.38**

**Figure 2:**
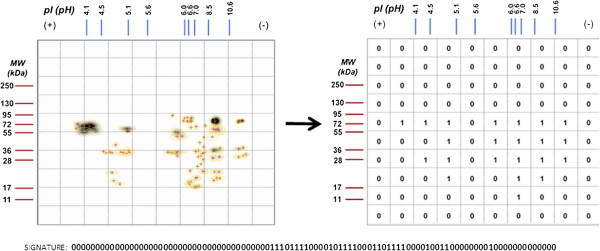
**Development of a multibinomial signature based in the arrangement of spots in a 2D-immunoblot image, which was divided in cells.** A "1" was placed in cells that had a one o more spots, while a "0" was placed, when there were not spots.

At time 0, the difference in spots number between the IgG and IgM was clear, with the IgG spots being almost zero, and the IgM spots numerous. The pattern of spots in the IgM response at was highly variable, and there were no 2 similar immunoblots. Identification of 4 T1 antigen by IgM, even before antigenic challenge (t = 0), was consistent with an innate humoral recognition mentioned above, nature of this recognition being through structural patterns of Toll-like receptors. Carbohydrates are the main structures recognized, but there are also reports where the natural IgM can recognize the carboxyl terminus of proteins [23]. There were 2 types of IgM found in normal conditions in the circulation in mice. Natural IgM is mainly secreted by B-1a CD5^+^ cells in the apparent absence of antigen stimulation, which constitutes most of the circulating IgM [24], whereas antigen-induced IgM is mostly produced by conventional B (B-2) cells only after antigen stimulation [25]. Thus antibodies detected at time 0 are called natural antibodies. Variability in the IgM response was manifested as a pattern of spots that became a multibinomial variable of 0 and 1, which could represent a signature of the IgM or IgG humoral immune responses (Figure 2); each signature is therefore a measure of this variability. Each mouse has an individual pattern of recognition, in this case to the tumor antigenic background, manifested in tumor size (Table 2). Thus each individual has a unique expression of genetically determined natural IgMs (in number and capacity of recognition). The possibility that this genetic determination does not recognize different tumors and their cells in the same way, or that some individuals recognize some kind of tumor better than others, is being investigated.

**Table 2:** **Tumor size in centimeters and volume of each female mouse, with classification according to size: small (1-1400 mm**^**3**^**), medium (1401-2800 mm**^**3**^**) and large (2801-4200 mm**^**3**^**)**

**Size of tumors**			
**N° female mice**	**Size (cm)**	**Volume (mm3)**	**Category**
**1**	1.8-1.5	2025	Medium
**2**	2.0-1.6	2560	Medium
**3**	1.0-0.7	245	Small
**4**	1.0-0.9	405	Small
**5**	0.8-0.6	144	Small
**6**	1.7-1.3	1436	Medium
**7**	2.2-1.9	3971	Large
**9**	1.2-2.0	600	Small
**10**	2.0-1.9	3610	Large
	**Average**	**1666.28**	
	**SD**	**1463.52**	

The cancer model we used did not allow us to establish a relation between a natural IgM response and resistance to tumor because this model was originally one for studying metastasis. Another type of mouse model, where diverse stages of cancer can develop will allow us to correlate natural IgM response with resistance.

The pattern recognition of IgM after inoculation of 4 T1 cells is not only caused by acquired immune response, but by the innate immune response. Some antigens that are recognized in the first week are also recognized by the same female at time 0. It is possible that these antigens in the first week, and sharing IP and MW with time = 0, are a product of an adaptive IgM in response to 4 T1 cells. The number of spots in Table 1 from the first to the fifth week for each mouse is the result of subtracting the antigens recognized at t = 0, which by coincidence were also detected in subsequent times of tumor development by isoelectric point and molecular weight determinations. Hence, in Table 1 the spots exclusively produced by the *innate* immune response (t = 0), separated from the spots produced exclusively by the *acquired* immune response (t = w1-5), antigens that are shared over time, were not considered exclusive. The significant number of antigens was recognized by IgM at the first week, from which we could use several antigens for early diagnosis because they are the first to be recognized by IgM and IgG immunoglobulins after antigen challenge with 4 T1 tumor cells. However, the antigens that are mainly recognized are the one that overlapped between t = 0 and w1 (Figure 1, Table 1). This overlap in the recognition of antigens between natural and adaptive IgM makes it difficult to use adaptive IgM as a diagnostic character of early immunodiagnostic. This difficulty could be overcome if technically one can distinguish structurally between natural IgM and adaptive IgM.

**Figure 3:**
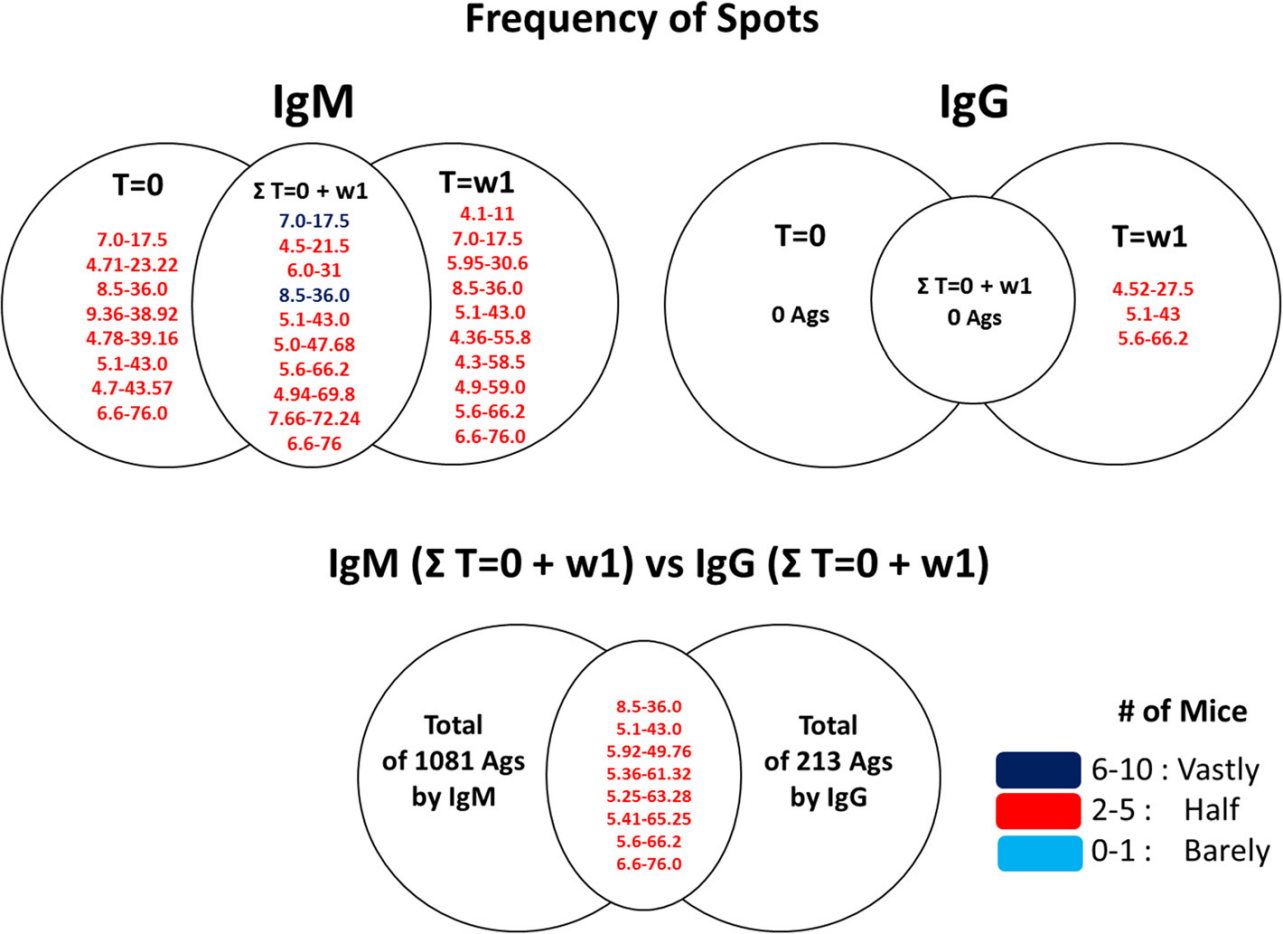
**Frequency of spots found in each 2D-immunoblot images of female mice in T = 0 and w1 of response mediated by IgG and IgM.** Figure shows the isoelectric point and molecular weigth of spots that are shared not only among images of each time (T = 0 and T = w1), but also among all female mice. Spots in red color are recognized "Half" and spots in blue heavy color are recognized "Vastly" by mice. Spots are recognized by 0 to 1 mice were categorized as "Barely", by 2 to 5 mice as "Half" and by 6 to 10 mice as "Vastly". Ags = antigens.

The number of spots in each 2D-immunoblot by immunoglobulin to the week of tumor development was estimated. Figure 3 gives frequency of spots of most of the antigens recognized, those half recognized and those barely recognized. The sum total of the spots of antigen recognition by natural IgM in the T = 0 was 563, and the frequency of spots for 8 antigens shared by all females mice at week 0. At week 1 (w1), the sum for all females was 518, and the frequency of spots related to 10 shared antigens. However, by comparing the frequency of antigen recognition spots at T = 0 and T = w1, this was low, with only 10 antigens shared between the two from a total of 1081. With respect to above mentioned IgM, the IgG response was reduced in the first week (T = w1), but the frequency of antigens recognized was similar between the IgG and IgM responses, being 3 from 183 and 10 from 518 shared antigens between individuals, respectively. The frequency of recognition of IgM and IgG against the same antigens shows that there are very few antigens detected by IgM or IgG shared among the individuals at time 0 and week 1, or between IgG and IgM. But the fact that IgM and IgG match some antigens in w1 that are the early antigen recognized indicates that they are particularly remarkable; this antigenic "selection" by the host could be used as a criterion to determine antigens in further work. This shared recognition reinforces the idea of using this system to find antigens in the human population that have dual recognition, not only IgM (natural or adaptive) but IgG, which can be help develop an early diagnostic test.

## Conclusion

IgM and IgG responses against 4 T1 cells vary with the time and between individuals. Spots variation in 2D pattern for natural IgM can be expressed as a binomial signature, which opens the way to correlate a particular pattern with resistance or susceptibility. The possibility of differentiating between adaptive IgM antibodies from the natural IgMs should allow the study of the adaptive IgM as a tool for early immunodiagnosis.

## Methods

### Mice and cell line

10 female BALB/c AnN mice 8 old-weeks were kept in the animal facilities at the Instituto de Investigaciones Biomédicas, UNAM. All experiments were carried out in accordance with the animal-welfare ordinance and approved by the Code of Ethics for Academic Staff of the Institute: ( http://www.biomedicas.unam.mx/_administracion/reglamentos_formatos/archivos_pdf/CodigoIIBO.pdf).

The 4 T1 tumor cell line was grown in RPMI +10% Fetal Bovine Serum +1 X of antibiotic-antimycotic mixture (streptomycin, ampicillin) in culture dishes (25 cm^2^) incubated at 37°C in air plus 5% CO_2_.

### Implantation of tumor cells

BALB/c mice was shaved on the abdomen and injected with 10 × 10^3^ 4 T1 tumor cells in 50 μL isotonic solution subcutaneously in a nipple of a mammary gland. The tumor was allowed to develop and grow for 30 days. Tumor size was measured after removal in 2 perpendicular dimensions with a Vernier caliper. Tumor size in cubic millimeters was calculated by the formula (a x b2)/2, where b was the smaller dimension of the tumor.

### Serum sampling

BALB/c mice were bled by the tail once per week for 6 weeks. The first bleeding was made before implanting 4 T1 tumor cells (time = 0) or pre-immune serum. After implantation of tumor cells, mice were bled and the sera weekly (w1, 2, 3, 4 and 5). Blood was incubated at 4°C for 30 min and centrifuged for storage at-20°C until use.

### 2D electrophoresis

For immunoblotting, proteins from 4 T1 cells were incubated with Abs from each serum of every mouse at different stages of tumor growth. A volume (100 μg) of 4 T1 cell proteins in 6 M urea, 50 mM DTT, 2% CHAPS, 2% ampholines pH 3-10 (Bio-Lyte), 0.001% bromophenol blue and MilliQ H_2_O up to 125 μL was used to hydrate the IPG strips (7 cm) in an immobilized pH gradient (pHi 3-10, Bio-Rad). To separate proteins from the 4 T1 cells or tumors in one dimension (isoelectric point), hydrated strips were placed in the Protean IEF Cell from Bio-Rad reaching a voltage of 4000 and 10,000 V-h, ~6 h.

After separation of proteins by isoelectric focusing, the strips were equilibrated with 2 washes for 10 min, the first in 6 M urea, 0.375 M Tris–HCl, pH8.8, 2% SDS, 20% glycerol, 2% (w / v) DTT, and the second in 6 M urea, 0.375 M Tris–HCl, pH8.8, 2% SDS, 20% glycerol, 2.5% (w / v) iodo-acetamide.

For the separation in a second dimension (2D) for molecular weight, the strip with separated proteins in one dimension was placed on an acrylamide gel (Mini-PROTEAN TGX Precast Gels, 10-20%, Bio-Rad) and electrophoresed in buffer 25 mMTris pH 8.3, 250 mM glycine, 0.1%SDS, at 100 V for 90 min in a chamber (Mini-Protean Tetra Cell, Bio-Rad).

The proteins separated on 2D were electrophoretically transferred to a nitrocellulose membrane using a wet transfer chamber (Mini Trans-Blot Cell, Bio-Rad) at 100 V, 1 h and 10 min; the buffer was Tris-Glycine-Methanol (25 mMTris pH 8.3, 250 mM glycine, 20% v/v methanol).

After transfer, the membrane was placed in 20 mL Sensitizer (12 mM HCl) for 5 min, and the solution was removed. It was placed in 20 mL CPTS dye ((Copper(II) phthalocyanine-3,4′,4″,4‴-tetrasulfonic acid tetrasodium salt (50 mg in 100 mL 12 mM HCl)), and was stirred strongly for protein staining. The dye was removed and the membrane washed with Sensitizer before being digitalized with a scanner (HP Scanjet G4050). The stained proteins were removed with Eraser solution (50 mL 0.2 M KCl + 40.8 mL 0.2 M NaOH, pH 12.5).

### ImmunoBlot

The nitrocellulose membrane was blocked with 30 mL 5% non-fat dry milk in PBS + Tween 20 (0.03%) at pH 7.4 and stirred overnight at 4°C. The next day the milk was removed and 5 mL skim milk in PBS-Tween and the primary Ab mouse serum (1:250) were placed and stirred for 4 h at room temperature. Four washes each for 10 min each of 20 mL PBS-Tween were use.

Bound antibodies were detected by incubation (1 h at room temperature) with HRP-conjugated secondary antibody (goat anti-IgG or IgM mouse, ZIMED), diluted 1/2500 in PBS-Tween 20. Each was washed 4 times for 10 min with 20 mL PBS-Tween. Detection of second antibody binding was by incubation with DAB substrate (3, 3′-diaminobenzidine; 0.5 mg/mL; 10 min) and 10 μL hydrogen peroxide.

### Image processing

From duplicates of the resulting 2D-immunoblot images, a master image was created, which was analyzed by counting the total number of spots (Σspots) or the number of unique spots per week were counted. Exclusive spots were obtained by subtracting the antigens recognized at t = 0, which had coincidence in isoelectric point and molecular weight, and were also detected at subsequent weeks of tumor development. Immunoblots were digitalized using an HP Scanjet G4050 scanner with a resolution level of 300 DPI in a TIFF format. The TIF images were transformed to the Format 1.sc, required for analysis in PDQuest software (Bio-Rad). The images were transferred to Adobe Photoshop for counting of spots, identification of coordinates of each spot in the 2D-immunoblot, and the calculation of the perimeters of the spots, location of the coordinates and the centroids. All 2D-immunoblots were analyzed at the same settings of brightness, contrast, and color to minimize bias.

## Abbreviations

IgG: Immunoglobulin G; IgM: Imunoglobulin M; 2D: Two dimension; DTT: Dithiothreitol; CHAPS: 3-[(3-cholamidopropyl)dimethylammonio]-1-propanesulfonate; SDS: Sodium dodecyl sulfate; HCl: Hydrochloric acid; KCl: Potassium chloride; NaOH: Sodium hydroxide; ∑: Summation; t = 0: Time 0.

## Competing interests

The authors declare that they have no competing interests.

## Authors’ contributions

MDZ carried out aspects of the mouse experiments and 2D Immunoblots, reviewed the manuscript its scientific veracity. RH participated in the execution of the mouse studies and participated in all experiments. POS conceived, designed and analyzed the collective results of all the experiments, and wrote the manuscript. All the authors read and approved the final manuscript.

## Authors’ information

MDZ has a Doctoral fellowship from the Consejo Nacional de Ciencia yTecnología, México. RH is a Master of Science and has an academic position at the Instituto de Investigaciones Biomédicas. POS is a PhD in Biochemistry and is a Researcher at the Instituto de Investigaciones Biomédicas. All work at the Universidad Nacional Autónoma de México.
